# Dopamine Preferentially Inhibits NMDA Receptor-Mediated EPSCs by Acting on Presynaptic D_1_ Receptors in Nucleus Accumbens during Postnatal Development

**DOI:** 10.1371/journal.pone.0086970

**Published:** 2014-05-01

**Authors:** Liming Zhang, Poulomee Bose, Richard A. Warren

**Affiliations:** 1 Centre de recherche Fernand-Seguin, University of Montreal, Montreal, Canada; 2 Department of Physiology, University of Montreal, Montreal, Canada; 3 Department of Psychiatry, University of Montreal, Montreal, Canada; Institute for Interdisciplinary Neuroscience, France

## Abstract

Nucleus accumbens (nAcb), a major site of action of drugs of abuse and dopamine (DA) signalling in MSNs (medium spiny neurons), is critically involved in mediating behavioural responses of drug addiction. Most studies have evaluated the effects of DA on MSN firing properties but thus far, the effects of DA on a cellular circuit involving glutamatergic afferents to the nAcb have remained rather elusive. In this study we attempted to characterize the effects of dopamine (DA) on evoked glutamatergic excitatory postsynaptic currents (EPSCs) in nAcb medium spiny (MS) neurons in 1 to 21 day-old rat pups. The EPSCs evoked by local nAcb stimuli displayed both AMPA/KA and NMDA receptor-mediated components. The addition of DA to the superfusing medium produced a marked decrease of both components of the EPSCs that did not change during the postnatal period studied. Pharmacologically isolated AMPA/KA receptor-mediated response was inhibited on average by 40% whereas the isolated NMDA receptor-mediated EPSC was decreased by 90%. The effect of DA on evoked EPSCs were mimicked by the D_1_-like receptor agonist SKF 38393 and antagonized by the D_1_-like receptor antagonist SCH 23390 whereas D_2_-like receptor agonist or antagonist respectively failed to mimic or to block the action of DA. DA did not change the membrane input conductance of MS neurons or the characteristics of EPSCs produced by the local administration of glutamate in the presence of tetrodotoxin. In contrast, DA altered the paired-pulse ratio of evoked EPSCs. The present results show that the activation D_1_-like dopaminergic receptors modulate glutamatergic neurotransmission by preferentially inhibiting NMDA receptor-mediated EPSC through presynaptic mechanisms.

## Introduction

The nucleus accumbens (nAcb), which forms the ventral part of the striatum, has been proposed to serve as an interface between limbic and motor systems [1]. The nAcb receives glutamatergic innervation from the medial prefrontal cortex and other limbic structures, including the hippocampus and amygdala (for review see [2],[3],[4],[5]) and it also receives a dense dopaminergic (DA) input from midbrain ventral tegmental area (VTA). Glutamatergic and dopaminergic afferents have been found to converge on the same dendritic spines of medium spiny (MS) GABAergic projecting neurons in the nAcb [6],[7], [8],[9],[10]. This closed spatial relationship suggests a possible interaction between the glutamatergic and dopaminergic systems at the pre- and/or postsynaptic levels. Behavioral studies have shown that interactions between DA and glutamatergic synaptic transmission, particularly those mediated by NMDA receptors, play a key role in animal behaviors associated with the nAcb (see [11],[12]). Recent finding of D_1_/NMDA receptor complexes in striatal and hippocampal tissue indicates possible direct protein-protein interactions between D_1_ and NMDA receptors [13].

In the nAcb, expression of NMDA receptor-dependent long-term potentiation has been demonstrated ([14],[15],[16]) and plasticity within nAcb is thought to mediate instrumental learning processes and many aspects of drug addiction in which coincident activation of NMDA and dopamine D_1_ receptors is required ([17],[18],[19],[20]). The nAcb may thus constitute a locus where NMDA receptors promote drug reinforcement [21]. In addition, the nAcb appears to be involved in a number of functions such as motivation, attention and reward ([1],[22]) which are modulated by the mesolimbic dopaminergic system [23].

Despite the well-known role of nAcb dopaminergic innervation in the modulation of motivated behaviors, and the recent advances in the understanding of cellular and molecular aspects of dopaminergic and glutamatergic receptor interaction [24], little is known about the [25] interaction between glutamatergic and dopaminergic function in the nAcb during postnatal development. Recent studies ([26],[27]) have shown that there are important changes in glutamatergic neurotransmission from the day of birth throughout adulthood. Of particular interest is a change in the amplitude of the NMDA receptor-mediated excitatory postsynaptic current (EPSC) to AMPA receptor-mediated EPSC ratio which reaches its maximum toward the end of the second postnatal week [26] and decreases after that until adulthood [27]. In addition to changes in glutamatergic neurotransmission, it has also been found that postnatal development is accompanied by changes in the dopaminergic innervation [28] as well as the density([29], [30],[31],[32]) and expression ([33],[30],[34] ) of dopaminergic receptors.

The effects of dopamine on glutamatergic neurotransmission have been previously studied. Some studies reported that the activation of D_1_ receptors enhanced NMDA receptor-mediated EPSCs in dorsal striatal slices ([35],[36],[37],[25],[38]), while others reported that D_1_ receptor agonists attenuated NMDA EPSCs in MS striatal neurons in culture ([24],[39]). In the nAcb, some investigators reported that DA or D_1_ receptor agonists potentiate NMDA receptor-mediated EPSCs in slices ([40],[41]), while others reported no significant modulatory effects of DA on NMDA receptor-mediated EPSCs ([42],[43]. It has also been shown in nAcb slices, that activation of D_1_ receptors inhibit glutamatergic synaptic transmission by a presynaptic action ([44],[45],[46],[47],[43], [48]) but the presynaptic inhibitory effect of DA on EPSCs was only determined on AMPA/KA receptor-mediated EPSCs in the nAcb. A substantial effect of DA on pharmacologically isolated NMDA and AMPA/KA receptor-mediated EPSCs remains to be determined.

In a previous study, we showed that acetylcholine (Ach) presynaptically modulated AMPA/KA and NMDA receptors mediated EPSCs in a parallel fashion in the nAcb during postnatal development [49]. In an effort to clarify how the NMDA and AMPA/KA EPSCs might be affected in the nAcb by dopaminergic innervation, we investigated the effect of DA on NMDA and AMPA/KA excitatory synaptic transmission in this region. Our results demonstrate that DA depresses the excitatory input onto MS neurons by the activation of presynaptic D_1_-like receptors. While DA depressed the elicited AMPA/KA receptor-mediated EPSCs in MS neurons by 40% of the control, DA almost completely abolished NMDA receptor-mediated EPSC. The effects of DA on glutamatergic EPSCs remained constant throughout the first 3 postnatal weeks.

## Materials and Methods

### Slice Preparation

All animal procedures were conducted in strict accordance with the Guide to the Care and Use of Experimental Animals (Second edition) of the Canadian Council on Animal Care. Protocoles were approved by the Comité de Déontologie pour l’Expérimentation sur des Animaux at the Centre de Recherche Fernand-Séguin. One to 21-day-old (P1–P21) Sprague Dawley rat pups of either sex obtained from Charles River (St-Constant, QC) were used in the present experiments. P5 and younger pups were anaesthetized by hypothermia whereas P6 and older animals were anesthetized by inhalation of methoxyfluran vapor in a closed environment. Once anesthetized, animals were decapitated and their brain was quickly removed and transferred to chilled, oxygenated artificial cerebrospinal fluid (ACSF) in which NaCl had been replaced by equivalent osmolarity of sucrose and containing (in mM) sucrose 252 (NaCl 126 in standard ACSF); KCl, 3; NaH_2_PO_4_, 1.25; MgSO_4_ 7 H_2_O, 1.3; CaCl_2_, 2.5; NaHCO_3_, 26; and glucose, 10, and saturated with a gas mixture of 95% O_2_ and 5% CO_2_. 400 µm thick parasagittal slices comprising the nAcb were cut using a vibrating microtome (Campden Instruments). Slices were transferred to a submerged type of recording chamber and continuously superfused with standard ACSF at room temperature (20–22°C) at a rate of 1.5 ml/min. In some experiments neurons were recorded at a temperature of 30–32°C to identify any differences in observed effects due to temperature. The nAcb was visualized under a stereomicroscope (Leica Inc.) using the anterior commissure, the neostriatum, the septum and the ventricles as landmarks based on [50]. The slices were incubated for at least one hour before recording.

### Recording

Whole-cell configuration was achieved using the ‘blind’ patch-clamp technique [51]. Pipettes were pulled from thin wall borosilicate capillary glass on a P-87 micropipette puller (Sutter Instrument, Novato, CA, USA). The pipettes had a resistance of 3–5 MΩ when filled with a solution containing (in mM) potassium gluconate, 140; MgCl_2_, 2; CaCl_2_, 0.1; EGTA, 1.1; HEPES, 10; K_2_-adenosine trisphosphate (ATP), 2; guanosine trisphosphate (GTP), 0.5 and 0.3% neurobiotin. The pH was adjusted to 7.3 with KOH solution, and final osmolarity was 280±5 mosmol/kg. QX314 (5 mM; Alomone Laboratories, Jerusalem, Israel) was routinely added to the recording pipette solution to prevent voltage-sensitive Na^+^ channels from generating action potentials. In some experiments, QX314 was omitted to account for any differences in observed effects caused by it.

Whole-cell recordings were made with an Axoclamp 2B amplifier (Molecular Devices, Sunnyvale, CA, USA) in continuous single-electrode voltage-clamp mode. The output of the amplifier was fed into a LPF 200A DC amplifier/filter (Warner Instruments Corp., Hamden, CT, USA) and digitized at 5 to 10 kHz with a real-time acquisition system Digidata 1200 (Molecular Devices). Data acquisition was achieved using the pClamp 6.0 software (Molecular Devices). The recording pipette’s capacitance was optimally adjusted before whole-cell configuration was achieved. The resting membrane potential was measured just after rupturing the cell membrane and the offset potential, measured upon withdrawal of the electrode from the cell, was accounted for assuming that it drifted in a linear fashion with time from the start of the recording session. We did not correct for liquid junction potential, which for a pipette containing 140 mM potassium gluconate amounts for an additional potential shift of around −10 mV [52].

### Synaptic Stimulation and Drug Application

A monopolar tungsten stimulating microelectrode was placed towards the rostral pole of the nAcb on the slice superficial layer, 0.5–1.0 mm from the recording electrode. Excitatory postsynaptic currents (EPSCs) were evoked by 0.1 ms, 3 to 6 V cathodal pulses delivered at 15 sec intervals. In some experiments, paired-pulse stimulation separated by 50 ms were used to distinguish between pre- and postsynaptic mechanisms. In order to isolate glutamate receptor-mediated EPSCs, all experiments were performed in the presence of (−) bicuculline methiodide (BMI, 10 µM) in bath solution to block GABA_A_ receptor-mediated synaptic currents. BMI was applied 30 min before obtaining whole-cell configuration to ensure a complete diffusion in the slice tissue. In all experiments the EPSCs were recorded from online voltage-clamped potentials between −100 and +40 mV in 20 mV increment from a holding membrane potential of −70 mV. 10 additional neurons were clamped at −70 mV and EPSC s were recorded at this same holding potential without incrementing. Local application of glutamate (10 mM) onto nAcb was conducted using a patch pipette connected to a Picospritzer (General Valve Corp., Fairfield, NJ, USA) under differential interference contrast and infrared optics using pressure pulses of 15 psi lasting 5–10 msec.

The following pharmacological agents were applied through the superfusing ACSF: 6 cyano-7-nitroquinoxaline-2,3-dione (CNQX), (+)-2-amino-5-phosphonopentanoic acid (APV) and (−) bicuculline methiodide obtained from Tocris Cookson (Bristol, UK); dopamine HCl, S-(−)-5-amino-sulfonyl-N-[(1-ethyl-2-pyrrolidinyl)-methyl]-2-methoxybenzamide (sulpiride), (4aR-trans)-4,4a,5,6,7,8,8a,9-octahydro-5-propyl-1H-pyrazolo[3,4-g]quinoline [(−)-quinpirole hydrochloride], (±)-1-phenyl-2,3,4,5-tetrahydro-(1H)-3-benzazepine-7,8-diol (SKF-38393), R(+)-7-chloro-8-hydroxy-3-methyl-1-phenyl-2,3,4,5-tetrahydro-1h-3-benzazepine hydrochloride [(+)-SCH-23390] and clozapine obtained from Sigma-Aldrich (Oakville, Ontario, Canada). Most drugs were made up as 10 mM stock solutions in distilled water (dopamine on the day of use) and diluted with ACSF solution to final concentration just before addition to the perfusion medium. The same procedure was used for CNQX except that it was initially dissolved in dimethysulfoxide (DMSO, final concentration 0.1%). Antagonists were applied for at least 15 min before application of agonists.

### Current Measurements

Data analysis was done using Signal software (Cambridge Electronic Design, Cambridge, England). The amplitude of the evoked synaptic current was plotted as a function of voltage at two latencies following stimulation: one at the peak of the inward current recorded at −100 mV and another later one at a point when the fast inward current at −100 mV just decayed to base line. This latter point was usually close to the maximal amplitude of the late component as recorded in the presence of the AMPA/KA receptor antagonist CNQX and no postsynaptic current was observed at this point at a holding membrane potential of −100 mV under these conditions ([53],[54]). The maximal amplitude of the late component of the EPSC was usually observed at a holding membrane potential of −20 or −40 mV and traces showing maximal responses were used in the illustrations. The effects produced by dopaminergic agonist did not change during the postnatal period studied and data were pooled accordingly.

### Statistics

Statistical analysis was performed using Sigmastat software (Systat, San Jose, CA, USA) and the effects of dopaminergic compounds on evoked EPSCs were tested using paired *t-*test unless otherwise stated (p values of less than 0.05 were considered as statistically significant). All numerical data are expressed as mean ± standard error of the mean (S.E.M). Neurons that could not be unambiguously classified as MS cells using physiological and morphological parameters were excluded from statistical analysis.

## Results

Whole-cell voltage-clamp recording was obtained from 169 physiologically identified MS neurons ([55],[56],[49],[26]) in 82 slices from rat pups aged between P1 and P21. The membrane and firing characteristics of MS neurons were similar to those previously reported for animals of comparable age [56]. Seven neurons,(n = 7) were recorded at a temperature of 30–32°C with the local anaesthetic QX314 in the internal solution and another 8 neurons were recorded with no QX314 in the internal solution. This was done to account for any difference in observed effects due to temperature or the addition of QX314. In addition, 58 neurons filled with neurobiotin were examined under light microscopy and displayed features that have been previously attributed to MS neurons from animals of similar age [57]. All labelled neurons appeared to be located in the core region of the nAcb.

### Characteristics of Glutamatergic EPSCs

As previously described ([49],[26]), local electrical stimulation, in the presence of the GABA_A_ receptor antagonist BMI, evoked an EPSC in all recorded neurons. Typically, the evoked response consisted of a compound glutamatergic EPSC comprising of an early and a late component mediated respectively by AMPA/KA and NMDA receptors.

**Figure 1:**
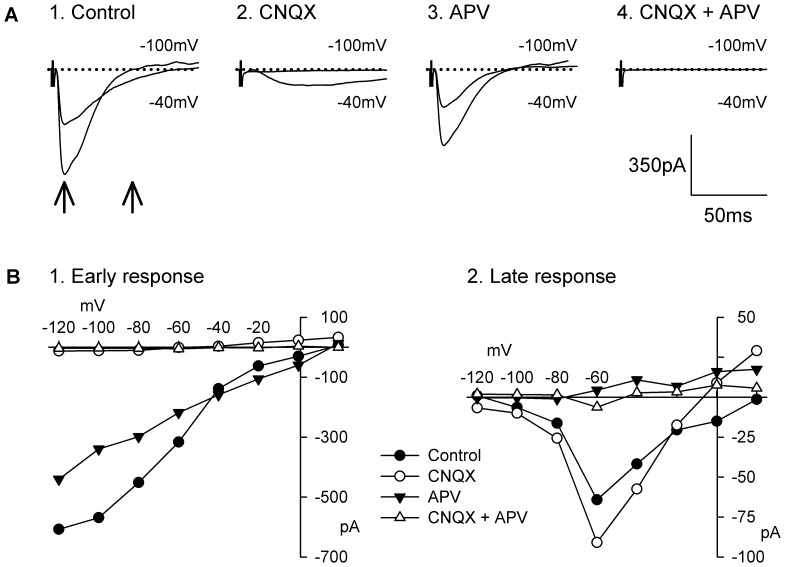
Nature of the EPSC evoked by local electrical stimulus in the presence of BMI (10 µM). **A**: current traces of the response evoked by single local electrical stimulus and recorded at holding membrane potentials of −40 and −100 mV before glutamatergic antagonists application (1.Control) and during superfusion with CNQX (20 µM; 2.CNQX), APV (50 µM) following CNQX wash out (3. APV) and CNQX and APV (4. CNQX+APV). Recordings were obtained in a MS neuron from a P20 animal. Current traces represent the average of 6 sweeps. **B**: Current-voltage relationship of the response recorded between −120 mV and 20 mV. The early component of the EPSC was measured 9 ms after the stimulus as indicated by the left vertical arrow in A. The late component was measured 53 ms after the stimulus as indicated by the right vertical arrow in A.

Figure 1 shows a representative example of an EPSC recorded in a preparation from a P20 animal on which specific glutamatergic antagonists were tested. During the control period (Fig. 1A panel 1), the early EPSC peaked 9 msec after the stimulus onset at a holding membrane potential of −100 mV and the response decayed to baseline within 45 msec. The current-voltage relationship of the early EPSC was close to linear and reversed at a membrane potential close to 0 mV (Fig. 1B panel 1). Bath application of the AMPA/KA receptor antagonist CNQX completely abolished the early component of the EPSC and there was virtually no residual postsynaptic current at all membrane potentials at the latency at which the early response was measured (Fig. 1A panel 2 and Fig. 1B panel 1).

The late component, measured after the early component had decayed, increased at membrane potentials between −100 and −40 mV, reaching its maximum usually at −40 mV or −20 mV (Fig. 1A and Fig. 1B panel 2). At more depolarized membrane potentials, it decreased and reversed polarity around 0 mV, a current-voltage relationship typical of NMDA receptor-mediated current. The addition of the NMDA receptor antagonist APV to the superfusing medium completely abolished the late EPSCs (Fig. 1A panel 3), demonstrating that it was mediated by NMDA receptors. There was no residual postsynaptic current in the presence of CNQX and APV, showing that glutamatergic EPSCs were effectively isolated by the addition of BMI to the superfusing medium (Fig. 1A panel 4 and Fig. 1B). CNQX and APV were tested together in 4 other neurons producing similar results. In addition, CNQX and APV were tested individually in 17 and 14 neurons respectively producing an inhibition of the early and late components of the response by 91±2% and 85±5%.

### Effects of Dopamine on AMPA/KA and NMDA Receptors Mediated EPSCs

The effects of dopaminergic agonists and antagonists were assessed at holding membrane potentials usually between −100 and +40 mV in steps of 20 mV. The AMPA/KA EPSC was measured at the peak of the early component of the EPSC at a holding membrane potential of −100 mV, when the amplitude of the late component was minimal (left vertical arrow in Fig. 1A panel 1) whereas the effects on NMDA EPSC was measured at a latency at which the early component recorded at a holding membrane potential of –100 mV had decayed (right vertical arrow in Fig. 1A panel 1).

**Figure 2:**
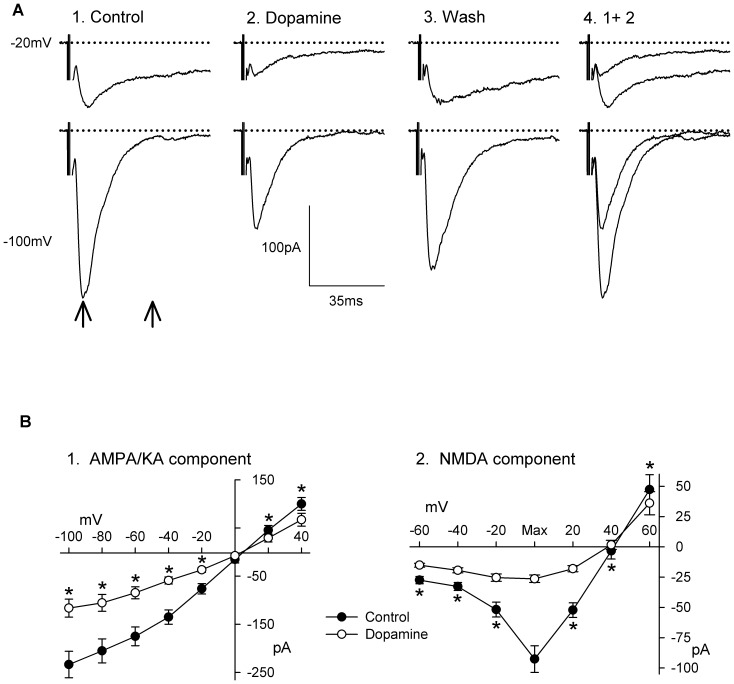
Effect of dopamine on the EPSC. **A**: Current traces of the response evoked by single electrical stimulus recorded at holding potentials of −20 mV (upper row) and −100 mV (lower row) before (1), during (2), and after (3) superfusion with dopamine (DA 50 µM). 4: the overlay of the responses before and during DA superfusion. Current traces represent the average of 6 sweeps. Recordings were obtained in a slice from a P7 animal. Left and right vertical arrows in 1 indicate where the AMPA/KA and NMDA responses were measured. **B**: Average current-voltage relationship of the AMPA/KA (n = 51; 1) and NMDA (n = 51; 2) responses recorded before and during superfusion with DA. The I_R_-V_m_ of the late response were aligned on the holding membrane potential at which the response was maximum before averaging (usually at −20 mV or −40 mV). Asterisks indicate a statistically significant difference between control and DA treatment at this holding membrane potential (Student’s *t*-test, *P*<0.05).

The addition of dopamine (50 µM) to the superfusing medium for 5–10 min (time course is shown below) resulted in a decrease in the amplitude of both AMPA/KA and NMDA components of EPSC. The decrease in EPSC amplitude occurred at all holding membrane potentials and a representative example of this effect is shown in Figure 2A. In this case, the NMDA and AMPA/KA components of the EPSC recorded at −20 mV and −100 mV (Fig. 2A) were respectively reduced by 70% and 41% during the application of DA. Similar inhibitory results were documented in 51 neurons whereas DA was found to produce no effects on the EPSC in only one neuron. The effects of DA were reversible and reproducible.

The effects of DA on the AMPA/KA and NMDA components of the EPSC as a function of holding membrane potential are summarized in Figure 2B. The amplitude of the AMPA/KA component was significantly reduced at all holding membrane potentials between −100 mV and +40 mV by 45 to 51% and that of the NMDA component by 57 to 73% between −60 mV and −20 mV. The effects of DA on the NMDA component were generally of larger magnitude than those observed on the AMPA/KA component. The inhibitory effect of DA on the NMDA component of the EPSC measured at its peak (−71±2.4%) was significantly larger than that on the AMPA/KA component measured at −100 mV (−51±2.4%; paired Student’s t-test, p<0.001, n = 51) resulting in an overall decrease in the NMDA to AMPA/KA response ratio. Indeed, we found that the NMDA to AMPA/KA component ratio was reduced by an average of 45±4% (from 0.47±0.04 to 0.26±0.03) in 42 neurons whereas it was increased by 24±5% (from 0.37±0.05 to 0.46±0.07) in 9 neurons. These results demonstrate that DA decreased the NMDA to AMPA/KA response ratio in a majority of neurons and that DA more effectively reduces NMDA than AMPA/KAR rather than decreasing them both equally.

**Figure 3:**
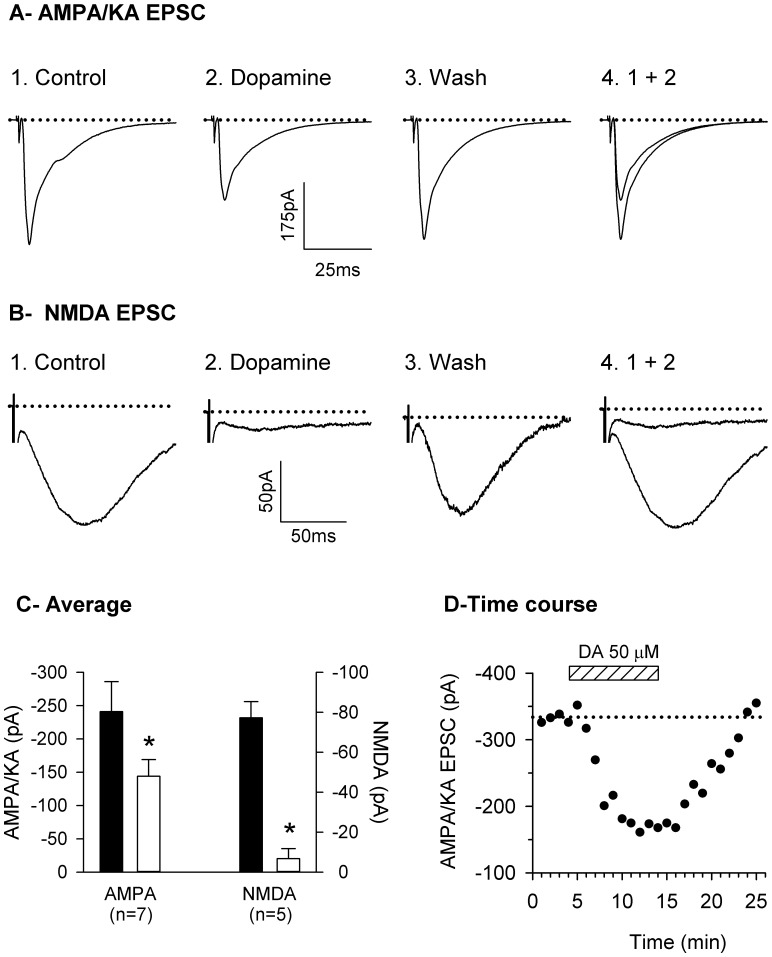
Effect of DA (50 µM) on pharmacologically isolated NMDA and AMPA/KA receptor-mediated EPSCs. **A**: Current traces of pharmacologically isolated AMPA/KA EPSC recorded with APV (50 µM) present in the superfusing medium before (1), during (2) and after (3) superfusion with dopamine at a holding membrane potential of −100 mV. 4: the overlay of the responses before and during DA superfusion. **B**: Current traces of pharmacologically isolated NMDA receptor-mediated EPSC recorded with CNQX (20 µM) present in the superfusing medium before (1), during (2) and after (3) superfusion with dopamine at a holding membrane potential of −20 mV. 4: the overlay of the responses before and during DA superfusion. Traces in A and B represent the average of 6 sweeps. **C**: average effect of dopamine on isolated NMDA and AMPA/KA receptor-mediated EPSCs. NMDA receptor-mediated EPSC was measured at the membrane potential the response was largest, −40 mV or −20 mV and AMPA/KA receptor-mediated EPSC was measured at a holding membrane potential of −100 mV. The left and right vertical axis are for the NMDA and AMPA/KA receptor-mediated EPSCs respectively. * Statistically different from control, Student’s paired t-test, *P*<0.001. **D**: Time course of the inhibitory effect (in percentage) of dopamine on AMPA/KA and on NMDA receptor-mediated EPSC shown in A and B respectively. Response was recorded every 15 s and each filled circle represent the average of 4 sweeps recorded over one minute period. The dashed rectangle represent the period during which DA (50 µM) was added to the superfusing medium, from 4 to 14 min.

To validate our experimental assumption that the effects of DA on compound EPSCs effectively represented the effects on AMPA/KA and NMDA receptor-mediated EPSCs, we studied the effects of DA on pharmacologically isolated AMPA/KA and NMDA mediated EPSC using APV (50:M) and CNQX (20:M), respectively. Figures 3 A and B show examples of the effect of DA on isolated EPSCs and, in agreement with what was observed in the compound EPSC, DA produced an inhibition of both AMPA/KA (−36%) and NMDA (−86%) EPSCs. The isolated APMPA/KA EPSC was reduced on average by 40% (n = 7) in the presence of DA whereas the NMDA EPSC was reduced by 90% (n = 5; Fig. 3C). In these experiments, DA produced an inhibition of the AMPA/KA receptor-mediated EPSC of a magnitude comparable to the effects observed on the compound EPSC whereas the inhibition of the NMDA receptor-mediated component was much larger. Figure 3D illustrates the time course of the effect of DA on the AMPA/KA and NMDA EPSCs shown in Figure 3A and 3B respectively. Both EPSCs decrease with a similar time course even though the inhibition of the NMDA EPSC was much larger than that of the AMPA/KA EPSC. The inhibition reached its maximum 6 or 7 min after DA superfusion was started and recovery of the response occurred after 10–12 min of DA wash out.

DA receptors’ expression and concentration have been shown to change significantly during postnatal development in the nAcb ([58],[59],[60],[31],[28]) and we tested the possibility that the inhibitory effect of DA changed concomitantly during the postnatal period. The inhibition produced by DA was on average slightly larger in younger than in older animals but the difference was not statistically significant as shown by an analysis of variance comparing the effects of DA on EPSCs between first, second and third postnatal weeks (p = 0.366 and p = 0.353 for the AMPA and NMDA components of the EPSCs respectively). There was also no significant correlation between the magnitude of the inhibitory effect of DA and postnatal age (p = 0.065 and p = 0.206 for the AMPA and NMDA components of the EPSCs respectively).

### Effects of Dopaminergic agonists and Antagonist

**Figure 4:**
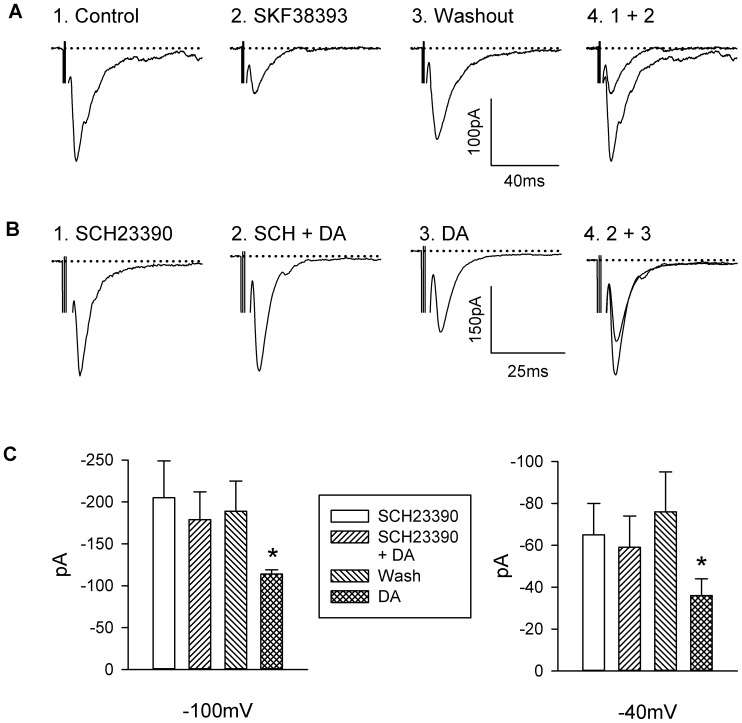
Effect D_1_-like receptor agonist and antagonist on dopaminergic inhibition of EPSC. **A**: Current traces of glutamatergic EPSC recorded before (1), during (2) and after (3) superfusion with SKF 38393 (10 µM) at a holding membrane potential of −100 mV. 4: the overlay of the responses before and during SKF 38393 superfusion. **B**: Current traces of glutamatergic EPSC recorded during superfusion with SCH23390 (1), with SCH23390 and DA (2) and with DA following the washout of SCH23390 (3) at a holding membrane potential of −100 mV. 4: the overlay of the responses during SCH23390 and DA and during DA. **C**: summary of the effect of DA in the presence or absence of SCH23390 at holding membrane potential −100 mV and −40 mV (n = 9). * Statistically different from SCH23390 and DA, Student’s paired t-test, *P*<0.01.

To identify the DA receptor subtype responsible for the inhibition of EPSCs, we examined the effects of D_1_- and D_2_-like receptor agonists and antagonists on stimulus-evoked EPSCs. Like DA, SKF 38393(10 µM), an agonist of D_1_-like DA receptors, decreased the amplitude of the AMPA/KA component of the EPSC by 43% at a holding membrane potential −100 mV (−304±131 pA in control and −173±76 pA, in SKF 38393, n = 5) and that of the NMDA component by 53% at a holding membrane potential −40 mV (−51±23 pA in control and −24±15 pA in SKF 38393). Figure 4A shows a representative example of the inhibitory effect of SKF 38393 on the AMPA/KA component of the EPSC.

In agreement with the effect produced by SKF38393, the D_1_-like receptor antagonist SCH 23390 (10 µM) reversibly antagonized the effect of DA (50 µM) on both AMPA/KA and NMDA components of the EPSC (Fig. 4B and 4C). Lower concentrations of SCH 23390 (1 or 5 µM) only partially blocked the inhibitory effects of DA (n = 20, data not shown).

In contrast, the D_2_-like receptor agonist quinpirole (10 µM) failed to inhibit the EPSCs (n = 4, not shown) whereas in the presence of the D_2_-like receptor antagonist sulpiride (10 µM), DA reduced the AMPA/KA and NMDA components of the EPSC respectively by 39±9% and 48±8% (p<0.001, n = 4). Clozapine (10 µM), an antagonist of D_2_-like dopamine receptors and of certain 5-HT receptors, also failed to block the inhibitory effect of DA which produced a reduction of the AMPA/KA and NMDA components of the EPSC respectively 60±12% and 76±10% (p<0.001, n = 9) in the presence of clozapine, further excluding the possibility of the involvement of D_2_-like receptors.

### Locus of the Dopaminergic Modulation of EPSCs

The inhibitory effects of DA on evoked EPSCs could involve pre- or postsynaptic mechanisms and we examined several characteristics of our current recordings in the presence or absence of DA to identify the site of action of DA.

**Figure 5:**
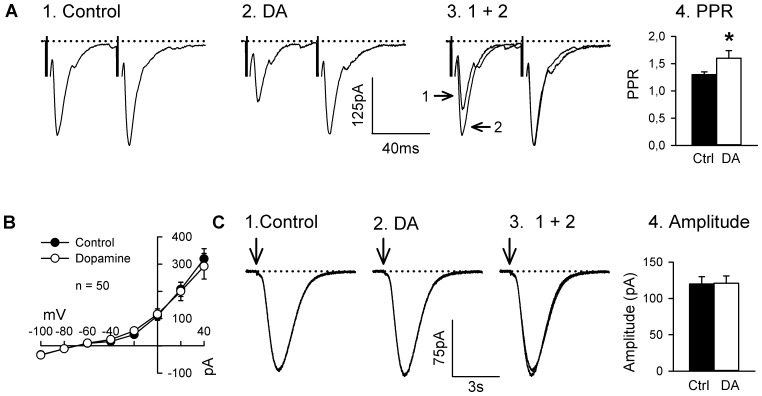
Locus of the effect of DA on EPSCs. **A**: Current traces of the responses evoked by a pair of single local electrical stimuli 50 ms apart at holding membrane potentials of −100 mV to record the AMPA response before (1) and during (2) superfusion with DA (50 µM). 3: overlay of the responses before and during DA superfusion; the amplitude of the 2nd response in the presence of DA was scaled to match the amplitude of the 2nd response during control. Note that the 1st response proportionally decreased more than the 2nd response in the presence of DA and that there is no substantial changes in the time course of the EPSCs. Current traces represent the average of 6 sweeps and BMI (10 µM) was present in the superfusing medium throughout recording. Panel 4 (4. PPR) shows the average amplitude of the PPR from 12 neurons before and during superfusion with DA. * Statistically different from control, Student’s paired t-test, *P*<0.05. **B**: Average holding membrane current before and during superfusion with DA (50 µM). No statistically significant differences were found at any holding membrane potential. **C**: current traces of the response evoked by local pressure ejection of glutamate (10 mM; vertical arrow) from a patch pipette before (1) and during (2) superfusion with DA (50 µM) at a holding membrane potential of –100 mV in the presence of tetrodotoxin (1 µM) and BMI (10 µM). 3: the overlay of the responses recorded in panels 1 and 2. 4: amplitude of the peak response recorded at a holding membrane potential of −100 mV for 5 neurons before and during superfusion with DA. No statistically significant difference in the amplitude of the glutamate response during control and during DA superfusion (Student’s paired *t*-test, *P*>0.75, n = 5).

First, we used a paired-pulse protocol with a 50 ms interval between stimuli at holding membrane potentials of −100 mV to record the AMPA response before (1) and during (2) superfusion with DA (50 µM). In the presence of DA, the amplitude of both the first and second evoked EPSCs decreased, but the first response decreased to a larger extent and the paired-pulse ratio (PPR; 2nd EPSC amplitude/1st EPSC amplitude) significantly increased, thus suggesting presynaptic mechanisms (Fig. 5A). Second, we observed that the addition of DA to the superfusing medium did not change the holding membrane current at holding membrane potential between −100 and +40 mV (Fig. 5B), suggesting that DA produced no change in input resistance. Third, DA produced no change in the decay time constant (τ) of the evoked EPSC measured by fitting a single exponential to the pharmacologically isolated AMPA/KA response at holding membrane potential of −100 mV (10.94±1.8 ms and 12.64±3.1 ms during control and during DA superfusion, respectively, paired Student’s t-test, *P*>0.25, n = 7). Fourth, we found that DA produced no effects on the response evoked by pressure ejection of glutamate (10 mM) in the vicinity of MS neurons in the presence of Tetrodotoxin (TTX 1 µM; Fig. 5 C). Together, these results show that, under the present experimental conditions, DA produced no detectable postsynaptic effects in nAcb MS neurons and that the present results reflect an action on presynaptic D_1_-like receptors.

### Effects of QX314 on Dopamine Modulation of Glutamatergic EPSCs

**Figure 6:**
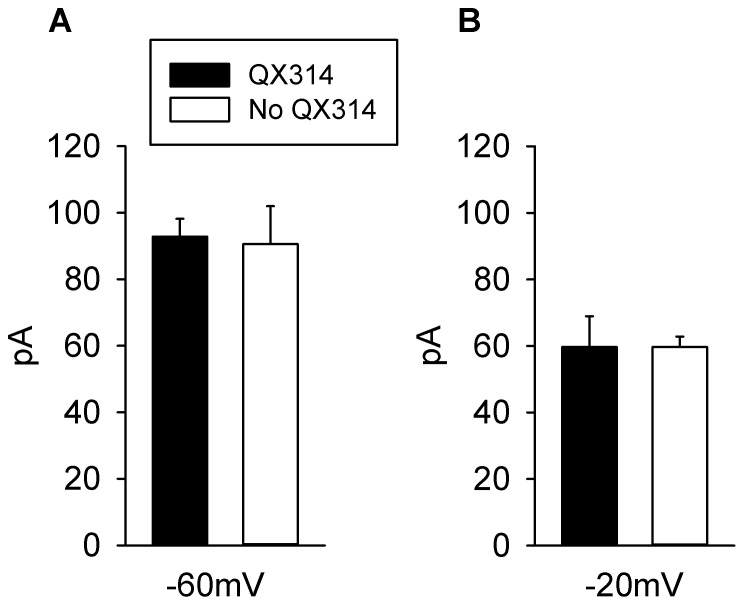
Effects of QX314 on Dopamine modulation of Glutamatergic EPSCs. **A**: Comparison of the average change in amplitude of the early component of the glutamatergic EPSCs (in pA) measured at −60 mV, on superfusion of DA in the presence and absence of QX314. The solid bar represents the average response in 80 neurons (n = 80), in the presence of QX314 and the dashed bar represents the average of 8 neurons (n = 8) in the absence of QX314. No statistically significant difference was found between the two groups (Paired Student’s t test p<0.050, p = 0.471). **B**: Comparison of the average change in amplitude of the late component of the glutamatergic EPSCs (in pA) measured at −20 mV, on superfusion of DA in the presence and absence of QX314. The solid bar represents the average of 70 neurons (n = 70), in the presence of QX314 and the dashed bar represents the average of 7 neurons (n = 7) in the absence of QX314. No statistically significant difference was found between the two groups (Paired Student’s t test p<0.048, p = 0.500).

In a previous study [49], we found that postsynaptic action mediated by muscarinic receptor was observed only when QX314 was omitted from the recording pipette. In addition, QX-314 is also known to inhibit G-protein-gated K^+^ conductance which could possibly lead to blocking dopaminergic postsynaptic effects on K^+^ conductance. To delineate the effects of QX314 on DA modulation of the early and late components of the glutamatergic EPSCs, 15 additional neurons were recorded with no QX314 in the recording pipette solution. The early component measured at −60 mV, was inhibited by DA by an average of 92.80±5.35 pA (n = 7) (Fig. 6 A) and was found to be statistically insignificant in comparison to the average inhibition by DA in the presence of QX314 (Paired Student’s t test, p<0.050,p = 0.471). The late component measured at −20 mV, was inhibited by DA by an average of 59.65±3.14pA (n = 6) (Fig. 6 B) and was found to be statistically insignificant in comparison to the average inhibition by DA in the presence of QX314 (Paired Student’s t test, p<0.048,p = 0.500). Thus it can be concluded, that the addition of QX314 in the recording pipette solution does not affect the inhibition produced by DA on the glutamatergic EPSCs.

Additionally, 15 neurons were recorded at a temperature of 30–32°C. Both the early and late components, measured at this temperature failed to produce any statistically significant difference in the inhibition produced by DA (not shown).

## Discussion

We found that DA inhibited glutamatergic EPSCs in MS neurons recorded in an *in vitro* slice preparation. Whereas both AMPA/KA and NMDA receptors-mediated components of the EPSCs were significantly inhibited by DA, the inhibition of the NMDA receptor-mediated component was much more pronounced than that of the AMPA/KA receptor-mediated component. Pharmacological evidence suggests that dopaminergic inhibitory effects were mediated by the activation of presynaptic D_1_-like receptors and that D_2_-like receptors were not involved. These results are in general agreement with previous studies showing that DA exerted a D_1_-like receptors mediated depression of excitatory synaptic response in the nAcb ([44],[61],[62],[63],[41],[42], [48]). To our knowledge, it is the first study directly comparing the effects of DA on AMPA/KA and NMDA receptors-mediated components of the ECSCs showing that DA exerted a stronger inhibition of NMDA than AMPA/KA receptor-mediated EPSCs. The latter finding supports the idea that there is direct D_1_-NMDA receptors interaction at cellular level ([64],[65],[66],[67],[68]). In addition, a recent study [69] suggests a dopamine triggered heterosynaptic plasticity mechanism, likely mediated presynaptically located D1 recetors and expressed by presynaptic inhibition of GABA release.

Most studies that have examined the effects of DA on glutamatergic neurotransmission in the nAcb have found a D_1_-like receptors-mediated depression of excitatory responses ([44],[61],[62],[48],[42],[43]) in agreement with our results. *In vitro* experiments have shown that this inhibition was mediated by presynaptic dopaminergic receptors [43], [45], [47], [62] located on glutamatergic terminals and are consistent with the demonstration that DA inhibited glutamate release in nAcb [70].

DA receptors [71] are distributed on both presynaptic glutamatergic terminals and postsynaptic membrane of MS neurons in the nAcb [28], [59], [60], [72]. Therefore, DA may have exerted its inhibitory action on EPSCs by acting at pre- and/or postsynaptic mechanisms. We examined the changes in PPR produced during DA application as an indication of pre- or postsynaptic mechanism. We found that bath application of DA greatly reduced the EPSCs but the inhibition was less on EPSC_2_ than on EPSC_1_ resulting in an increase in PPR, suggesting that DA acted presynaptically to reduce the probability of glutamate release from presynaptic terminals [73], [74]. We have also found that DA did not change membrane conductance nor that it affected the extrinsic glutamate-induced currents also arguing in favor of presynaptic mechanisms [74], [74]–[77]. This conclusion is also consistent with that of previous investigators studying DA inhibition on EPSCs in the nAcb [42], [44], [45], [48], [62] as well as in the nucleus of the solitary tract [74], the subiculum [78], the supraoptic nucleus [79] and the parabrachial nucleus [80].

In other studies, DA has also been shown to produce postsynaptic effects in MS neurons [38], [44], [81]–[85] but we and others [41], [47], [48], [61]–[63] failed to observe postsynaptic effects. In the present study, we added QX314 to the recording pipette solution in order to prevent voltage-sensitive Na^+^ channels from generating action potentials. QX314 as well as other local anaesthetics have been shown to interfere with G protein-coupled receptors as well as with other second messenger systems [86]–[90] and it is likely to have occluded the expression of dopaminergic postsynaptic effects. QX314 is also known to interfere with cholinergic muscarinic receptors [91], [92]. In a previous study [49] we found that postsynaptic action mediated by muscarinic receptor was observed only when QX314 was omitted from the recording pipette. In addition, QX-314 is also known to inhibit G-protein-gated K^+^ conductances [93]–[98] and this may have also occluded dopaminergic postsynaptic effects on K^+^ conductance reported by others [85]. The absence of dopaminergic postsynaptic effects in our preparation allowed us to isolate presynaptic mechanisms and to demonstrate a larger D_1_ receptor-mediated inhibitory effect on NMDA than on AMPA/KA EPSC.

The D_1_-like receptor agonist SKF 38393, but not the D_2_-like receptor agonist quinpirole, mimicked the action of DA. Consistent with these results, the D_2_-like antagonist sulpiride failed to inhibit the depressive effect of DA and the D_2_-like receptor antagonist/antipsychotic drug clozapine as well. The D_1_-like receptor antagonist SCH 23390 completely block the action of DA at a concentration of 10 µM, and only partially at 1 or 5 µM. These results are in agreement with previous studies [99].

The major finding of the present study is that the activation of D_1_-like receptors preferentially inhibited NMDA receptor-mediated EPSCs in the nAcb *in vitro*. Whereas several studies have found, in agreement with the present results, that dopaminergic agonist inhibited glutamatergic EPSCs in the nAcb, none compared the effects of dopaminergic agonists on NMDA and AMPA/KA receptor-mediated EPSCs and generally only compound excitatory postsynaptic responses were recorded at holding membrane potentials (usually −80 or −90 mV) at which no significant NMDA receptor-mediated currents are present [42]–[44], [48], [61], [62]. Only Harvey and Lacey [63] recorded pharmacologically isolated NMDA receptor-mediated EPSCs while holding the membrane potential at −50 mV in addition to recording compound EPSCs while voltage clamping the cells between −80 and −90 mV. They found that DA (30 µM) produced an inhibition of NMDA receptor-mediated EPSCs of 51±3.1% and, although DA produced also an inhibition of the compound EPSC, they did not report the magnitude of these effects. In a previous study [62] they reported that DA (30 µM) produced an inhibition of the compound EPSC of 56±11%, comparable to the effects produced on isolated NMDA receptor-mediated EPSCs reported in their former study.

The larger effect of DA on NMDA receptor-mediated EPSC may be due to a higher affinity of glutamate for AMPA/KA receptors than for NMDA receptor. In that case, the decrease in glutamate release produced by DA would have resulted in a smaller decrease of AMPA/KA receptor-mediated EPSC than the NMDA receptor-mediated EPSC. However, NMDA receptors have a much higher affinity for glutamate than do AMPA receptors and it has been proposed that the concentration of glutamate achieved in the synaptic cleft may often be sufficient to activate NMDA, but not AMPA receptors [100], [101].

There is evidence indicating that glutamatergic and dopaminergic afferents often synapse in close apposition on the same MS neuron spine [8], [102], [103] suggesting that the glutamatergic and dopaminergic systems interact in modulating MS neurons at the dendritic spines level [28]. Several studies have shown that D_1_ receptors have extensive functional interactions with NMDA receptor and the larger inhibition of the NMDA receptor-mediated EPSC could be the results of these interactions. This proposal is consistent with the findings that dopamine D_1_ receptors modulated NMDA receptor-mediated EPSCs through direct protein-protein interactions in cultured striatal and hippocampal neurons [24], [39]. It was found that two regions in the D_1_ receptor carboxyl tail are directly and selectively coupled to NMDA glutamate receptor subunits NR1-1a and NR2A and that through these interactions, D1 receptor agonists could selectively inhibit NMDA receptor-mediated currents through a PKA/PKC independent pathway [24]. The D_1_ receptor agonist SKF 81297 produced a decrease in the number of NMDA receptors expressed on the cell surface which could explain the observed D_1_ postsynaptic modulation of NMDA currents without changes in membrane conductance.

It was demonstrated that NMDA receptor has physical associations with D_1_ receptor and provided ultrastructural evidence that D_1_ receptors in the nAcb have subcellular distributions supporting intracellular co-trafficking with NR1 subunit of NMDA receptor [65]. In agreement with these findings, another group Tong and Gibb [68] found that, in an acute brain slice of neonatal P7 rat striatum, the D_1_ receptor agonist SKF-82958 significantly decreased the NMDA receptor-mediated current (−34%) produced by the exogenous application of NMDA in patch-clamp whole-cell recordings and that this inhibition was blocked by the intracellular application of a dynamin inhibitory peptide (QVPSRPNRAP) suggesting that a tyrosine kinase-dependent alteration of NMDA receptor trafficking underlies D_1_ dopamine receptor down-regulation of NMDA receptor currents in MS neurons of neonatal rats. Alternatively, Cui and group [66] found that DA and several D_1_ ligands (including D_1_ agonist SKF38393 and D_1_ antagonist SCH23390 used in the present study) could act as voltage-dependent, open channel blockers for NMDA receptor, regardless of whether they are agonists or antagonists for D_1_ receptor, suggesting that the direct inhibition of NMDA receptors by dopamine D_1_ receptor ligands could be due to the channel pore block rather than or in addition to receptor-receptor interaction.

We found that the inhibitory effects of DA on both AMPA and NMDA receptor-mediated EPSCs remained constant throughout the first 3 postnatal weeks despite the fact that there are important changes in the dopaminergic innervation of the nAcb during that period [28] as well as changes in the density [29], [30], [32], [104] and expression [30], [33], [34] of dopaminergic receptors. It has been shown that density of D_1_-like receptor increases from P7 to P28, then declines by 20–40% after P35 to remain unchanged until P60. Our observation did not parallel these changes in receptor density. One possibility is that changes in receptor density occur primarily on MS neurons themselves whereas there is little or no change in the receptor density on glutamatergic terminals. However, in agreement to our observations, it has been reported that there is indeed a D1 receptor mediated NMDA inhibition in young juvenile rats whereas an increase in D1 mediated excitability caused by NMDA on MSN neurons was observed in young adult rats [105]. Our finding thus suggests a possible mechanism by which DA exerts its effects on MSNs, however the circuitry of specific projection systems to and from the nACb need to be identified to understand their contribution in shaping the final output of the nAcb.
